# Study of single nucleotide polymorphisms of *FBW7* and its substrate genes revealed a predictive factor for paclitaxel plus cisplatin chemotherapy in Chinese patients with advanced esophageal squamous cell carcinoma

**DOI:** 10.18632/oncotarget.9736

**Published:** 2016-05-31

**Authors:** Ying Liu, Shu Ning Xu, Yong Shun Chen, Xiao Yuan Wu, Lei Qiao, Ke Li, Long Yuan

**Affiliations:** ^1^ Department of Medical Oncology of Henan Cancer Hospital, Zhengzhou University Affiliated Cancer Hospital, Zhengzhou, Henan, China; ^2^ Department of Radiation Oncology of Henan Cancer Hospital, Zhengzhou University Affiliated Cancer Hospital, Zhengzhou, Henan, China; ^3^ Department of Surgical Oncology of Henan Cancer Hospital, Zhengzhou University Affiliated Cancer Hospital, Zhengzhou, Henan, China

**Keywords:** paclitaxel, esophageal squamous cell carcinoma, chemotherapy, single nucleotide polymorphisms, FBW7

## Abstract

Paclitaxel plays a major role in the treatment of advanced esophageal squamous cell carcinoma. However, there is no biomarker that could be used to predict the clinical response of paclitaxel. This work was conducted to investigate the association of genetic polymorphisms in *FBW7* and its substrate genes and the clinical response of paclitaxel. Patients with advanced esophageal squamous cell carcinoma were treated with paclitaxel 175 mg/m^2^ over 3 hours day 1 and cisplatin 75 mg/m^2^ day 1, every 3 weeks. The genotypes of 11 *FBW7* and its substrate gene polymorphisms were determined by polymerase chain reaction–restriction fragment length polymorphism (PCR-RFLP) method. Statistical analysis revealed that patients with *mTOR* rs1057079 AG (OR_adjusted_: 4.59; 95% CI: 1.78-11.86) genotype had significant correlation with the clinical response of paclitaxel when compared with AA genotype after adjustment for sex, age, and chemotherapy cycle. The median progression-free survival (PFS) of patients with advanced ESCC who received paclitaxel plus cisplatin (TP) as first-line treatment is 14.3 months (95% CI: 9.0-19.60 months). The median PFS (mPFS) of AG genotypes and AA genotypes in *mTOR* rs1057079 were 17.31 months (95% CI: 15.9-18.67 months) and 9.8 months (95% CI: 8.58-11.02 months) (*p*=0.019), respectively.

## INTRODUCTION

Esophageal cancer is one of the most common and fatal malignancies in the world [1]. More than two-thirds of patients diagnosed with esophageal cancer had unresectable disease. Patients with unresectable or metastatic esophageal cancer have a particularly dismal prognosis, with an overall survival (OS) of 3.0-8.0 months [2]. Esophageal squamous cell carcinoma (ESCC) is the predominant histologic type (90%-95%), while the incidence of esophageal adenocarcinoma remains extremely low in China [3]. So far, no standard regimen is recommended for advanced or metastatic ESCC. Taxol, also known as paclitaxel, has been recognized as an anti-microtubule agent that stabilizes microtubule structure within the cells, causing mitotic arrest and apoptosis [4, 5]. Paclitaxel has been used for the treatment of various types of solid cancers, showing response rates in the range of 30-40% when used alone [6] and 43-56.5% when used in combination with cisplatin [7, 8] in ESCC. Notably, the clinical responses are entirely different among patients with advanced ESCC, which implicates that individual genetic variation could be a significant factor for drug sensitivity. It has been reported that single nucleotide polymorphisms (SNPs) have association with radiation and chemotherapy dependent pathways. A recent study [9] reported that FBW0\7-null colon cancer cells were more resistant to taxol-induced cell death than the wild type cells. Similar results were also observed in ovarian cancer cell lines with naturally occurring F-box and WD repeat domain-containing 7 (FBW7) mutations [10]. It has been determined that the specific substrates of FBW7 consist of cyclin E [11,12], c-Myc [13,14], c-Jun [15,16], Notch [16–18], presenilin [19], Mcl-1 [20], sterol regulatory element-binding proteins (SREBP) [21,22], mTOR [23], Kruppel-like factors (KLFs) [24,25], c-Myb [26], and Aurora A [27]. Proteins encoded by different SNPs of these *FBW7* substrate genes are frequently overexpressed in a variety of human cancers. Thus, we detected SNPs of *FBW7* and its substrate genes of patients with unresectable or metastatic ESCC who received TP as first-line chemotherapy to investigate the association between the efficacy of TP and the SNPs of *FBW7* and its substrate genes and to develop a predictive biomarker for identifying patients that would benefit from paclitaxel therapy.

## RESULTS

### Characteristics of the study population

From January 2012 to January 2015, 134 patients with ESCC met these criteria, and were recruited to receive the study treatment in Henan Cancer Hospital. Among them, 123 patients were evaluated for toxicity and efficacy, which includes 86 men (69.9%) and 37 women (30.1%) with a median age of 62 years (range: 35-75 years). All patients had metastatic disease, which included lymph nodes, mediastinum, lung, liver, adrenal grand, bone and soft tissues. In total, 33 patients (26.8%) received esophagectomy; 22 patients (17.9%) received radiotherapy. Baseline characteristics are summarized in Table 1. The median total duration of treatment was 4 cycles (2-6 cycles). Among the 123 patients, CR was not observed, 57 patients achieved PR and 39 patients experienced PD. The objective response rate (ORR) and the disease control rate (DCR) of patients with advanced ESCC treated with TP as first-line chemotherapy are 46.3% and 68.3%, respectively.

**Table 1:** Baseline patient characteristics

Characteristic	TP group N=123	
	No.	%
Sex		
Male	86	69.9
Female	37	30.1
Age, years		
Range	35-75	
Median	62	
ECOG Performance status		
0	78	63.4
1	27	22.0
2	18	14.6
Metastases		
Lymph nodes	92	74.8
Mediastinum	70	56.9
Lung	38	30.1
Liver	15	12.2
Bone	10	8.1
Others	11	8.9
Esophagectomy		
Yes	33	26.8
NO	90	73.2
Radiotherapy		
Yes	22	17.9
NO	101	82.1

Abbreviations: TP, paclitaxel plus cisplatin; ECOG: Eastern Cooperative Oncology Group

### Association of 11 *FBW7 and its* substrate gene polymorphisms with efficacy of paclitaxel

The genotype distributions of the 11 selected SNPs of *FBW7* and its substrate genes and the efficacy of paclitaxel are shown in Table 2. The results of unconditional logistic regression analysis of the genotypes revealed that the patients with *mTOR* rs1057079 AG (OR_adjusted_: 4.59; 95% CI: 1.78-11.86) genotype had significant correlation with the clinical response of paclitaxel when compared with AA genotype after adjustment for sex, age, and chemotherapy cycle. However, no significant correlations were observed between the other 10 genotypes examined and the clinical response of paclitaxel.

**Table 2:** Genotypes of 11 SNPs of *FBW7* and its substrate genes and their association with efficacy of paclitaxel

Gene (Genotypes)	PD+SD(%)	PR(%)	*χ^2^*	*OR*(95%*CI*)^a^	*P*^a^
rs210940(*myb*)					
CC	22(36.7)	14(26.4)	reference		
CT	32(53.3)	29(54.7)	2.42	2.70(0.70,10.35)	0.15
TT	6(10.0)	10(18.9)	0.68	1.07(0.42,2.74)	0.88
rs7435589(*myb*)					
AA	27(45.0)	22(42.3)	reference		
AG	28(46.7)	24(46.2)	0.02	0.89(0.37,2.12)	0.79
GG	5(8.3)	6(11.5)	0.33	1.36(0.33,5.62)	0.67
rs1057079(*mTOR*)					
AA	47(77.0)	27(50.9)	reference		
AG	14(23.0)	25(47.2)	7.58	**4.59(1.78,11.86)**	**<0.01**
GG	0(0.0)	1 (1.9)	0.00		1.00
rs17036508(*mTOR*)					
TT	54(90.0)	45(86.5)	reference		
TC	6(10.0)	7(13.5)	0.32	0.89(0.24,3.27)	0.86
rs2536(*mTOR)*					
TT	53(86.9)	46(83.6)	reference		
TC	7(11.5)	9(16.4)	0.52	1.47(0.46,4.74)	0.52
CC	1(1.6)	0(0.0)	0.00		1.00
rs2295080(*mTOR)*					
TT	40(76.9)	28(59.6)	reference		
TG	9(17.3)	16(34.0)	3.71	2.52(0.88,7.23)	0.08
GG	3(5.8)	3(6.4)	0.18	1.48(0.23,9.73)	0.68
rs3124591(*NOTCH1*)					
TT	48(78.7)	43(81.1)	reference		
TC	7(11.5)	7(13.2)	0.62	0.73(0.16,3.26)	0.68
CC	6(9.8)	3(5.7)	0.04	1.07(0.31,3.72)	0.92
rs10521(*NOTCH1*)					
AA	49(80.3)	43(78.2)	reference		
AG	10(16.4)	11(20.0)	0.20	2.25(0.18,27.78)	0.52
GG	2(3.3)	1(1.8)	0.37	4.41(0.29,66.09)	0.28
rs9642880(*c-Myc)*					
GG	26(42.6)	30(56.6)	reference		
GT	9(14.8)	8(15.1)	0.22	0.99(0.30,3.24)	0.99
TT	26(42.6)	15(28.3)	2.72	0.48(0.19,1.23)	0.12
rs9902941(*SREBF2*)					
CC	54(88.5)	49(92.5)	reference		
CT	6(9.8)	4(7.5)	0.21	0.88(0.18,4.29)	0.88
TT	1(1.6)	0(0.0)	0.00		1.00
rs7685296(*FBW7*)					
CC	23(37.1)	21(41.2)	reference		
CT	6(9.7)	8(15.7)	0.38	1.06(0.28,4.06)	0.93
TT	33(53.2)	22(43.1)	0.59	0.89(0.36,2.22)	0.80

a Adjusted for sex, age, and chemotherapy cycle

### Association of 11 *FBW7* and its substrate genes polymorphisms with situation of adverse events

The genotype distributions of the 11 selected SNPs of *FBW7* and its substrate genes and situation of adverse events after paclitaxel plus cisplatin chemotherapy are shown in Table 3. The results of unconditional logistic regression analysis of the genotypes in *myb* rs7435589 revealed that, compared with AA genotype, the individuals with AG genotype (OR adjusted: 2.86; 95% CI: 1.28-6.39) had significant correlation with the adverse events after adjustment for sex, age, and chemotherapy cycle. Similarly, the genotype distributions of the SNPs results also showed that SNPs rs10521 in *NOTCH1* had significant difference. Compared with AA genotype, the individuals with AG (OR_adjusted_: 3.23; 95% CI: 1.07-9.69) genotype showed significant correlation with the adverse events after adjustment for sex, age, and chemotherapy cycle. However, no significant differences in other genotypes were observed between the SNPs genetic variants with the adverse events.

**Table 3:** Genotypes of 11 SNPs of *FBW7*and its substrate genes and their associations with adverse events

Gene (Genotypes)	0^b^(%)	1^b^(%)	2^b^(%)	*χ^2^*	*OR*(95%*CI*)^a^	*P*^a^
rs210940(*myb*)						
CC	5(28.6)	14(37.8)	17(27.4)	reference		
CT	5(35.7)	20(54.1)	36(58.1)	0.64	1.40(0.61,3.20)	0.42
TT	4(35.7)	3(8.1)	9(14.5)	0.01	0.96(0.30,3.09)	0.95
rs7435589(*myb*)						
AA	6(42.9)	24(64.9)	19(31.1)	reference		
AG	5(35.7)	11(29.7)	36(59.0)	6.52	**2.86(1.28,6.39)**	**0.01**
GG	3(21.4)	2(5.4)	6(9.8)	0.04	0.87(0.24,3.12)	0.83
rs1057079(*mTOR*)						
AA	11(73.3)	20(54.1)	43(69.4)	reference		
AG	4(26.7)	17(45.9)	18(29.0)	1.28	0.64(0.29,1.39)	0.26
GG	0(0.0)	0(0.0)	1 (1.6)	0.00		1.00
rs17036508(*mTOR*)						
TT	13(86.7)	33(91.7)	53(86.9)	reference		
TC	6(13.3)	3(8.3)	8(13.1)	0.05	1.14(1.14,1.83)	0.83
rs2536(*mTOR)*						
TT	14(93.3)	28(73.3)	57(90.5)	reference		
TC	0(0.0)	10(26.3)	6(9.5)	1.28	0.55(0.19,1.55)	0.26
CC	1(6.7)	0(0.0)	0(0.0)	0.00		1.00
rs2295080(*mTOR)*						
TT	9(81.8)	24(66.7)	35(67.3)	reference		
TG	1(9.1)	10(27.8)	14(26.9)	0.01	0.98(0.38,2.52)	0.97
GG	1(9.1)	2(5.6)	3(5.8)	0.19	0.69(0.14,3.51)	0.66
rs3124591(*NOTCH1*)						
TT	12(75.0)	31(86.1)	48(77.4)	reference		
TC	2(12.5)	4(11.1)	8(12.9)	0.17	1.27(0.41,3.97)	0.68
CC	2(12.5)	1(2.8)	6(9.7)	0.14	1.30(0.33,5.19)	0.70
rs10521(*NOTCH1*)						
AA	13(86.7)	34(89.5)	45(71.4)	reference		
AG	2(13.3)	3(7.9)	16(25.4)	4.38	**3.23(1.07,9.69)**	**0.04**
GG	0(0.0)	1(2.6)	2(3.2)	0.33	2.06(0.17,24.63)	0.57
rs9642880(*c-Myc)*						
GG	8(53.3)	20(54.1)	28(45.2)	reference		
GT	1(6.7)	5(13.5)	11(17.7)	1.07	1.83(0.58,5.79)	0.30
TT	6(40.0)	12(32.4)	23(37.1)	0.00	0.99(0.44,2.21)	0.92
rs9902941(*SREBF2*)						
CC	14(93.3)	32(86.5)	56(90.3)	reference		
CT	1(6.7)	3(8.1)	6(9.7)	0.37	1.53(0.39,6.03)	0.40
TT	0(0.0)	2(5.4)	0(0.0)	1.37	0.20(0.01,2.90)	0.24
rs7685296(*FBW7*)						
CC	9(60.0)	16(42.1)	19(31.7)	reference		
CT	2(13.3)	4(10.5)	8(13.3)	0.68	1.66(0.50,5.53)	0.40
TT	4(26.7)	18(47.4)	33(55.0)	2.80	2.01(0.89,4.59)	0.09

a Adjusted for sex, age, and chemotherapy cycle

b represents the number of adverse events, 0: no adverse events, 1:1-2 grade adverse events, 2:≥3 grade adverse events

### Haplotype analysis of 11 *FBW7* and its substrate gene polymorphisms

Haplotype analysis was performed with two SNPs (rs210940 and 7435589) across the *myb* gene, four SNPs (rs1057079, rs17036508, rs2536 and rs2295080) across the *mTOR* gene and two SNPs (rs3124591 and rs10521) across the *NOTCH1* gene. For each susceptibility analysis of a certain haplotype, all other haplotypes were taken as a reference. As a result, 4 haplotypes were generated after haplotype analysis of the 2 haplotypes across the *myb* and *NOTCH1* genes respectively; and for *mTOR* gene, 16 haplotypes were generated. By abnegating those haplotypes with frequency less than 3%, the effective results are shown in Table 4 to Table 6. As shown in Table 5, haplotype of A_rs1057079_C_rs17036508_T_rs2536_T_rs2295080_ can increase the risk of an infaust efficacy of paclitaxel [*OR* (95%*CI*): 3.16(1.45-6.87)]. On the contrary, haplotypes of G_rs1057079_C_rs17036508_T_rs2536_G_rs2295080_and A_rs1057079_C_rs17036508_T_rs2536_T_rs2295080_ can decrease the risk of an infaust efficacy of paclitaxel [*OR* (95%*CI*): 0.25 (0.07-0.96) and *OR* (95%*CI*): 0.12 (0.02-0.96)]. At the same time, neither of the haplotypes across the *myb* gene nor the *NOTCH* gene was associated with an increased or decreased infaust efficacy of paclitaxel risk (*p*>0.05).

**Table 4:** Haplotype analysis of the *myb* gene in subjects

Haplotype	PD+SD (%)	PR(%)	*χ^2^*	*OR*(95%*CI*)	*P*
C^1^A^2^	8(6.8)	13(12.7)	2.16	0.51(0.20,1.27)	0.14
C^1^G^2^	36(29.9)	35(33.5)	0.30	0.85(0.48,1.50)	0.58
T^1^A^2^	74(61.5)	55(52.7)	1.97	1.47(0.86,2.52)	0.16

1 rs210940 site,

2 rs7435589 site

**Table 5:** Haplotype analysis of the *mTOR* gene in subjects

Haplotype	PD+SD (%)	PR(%)	*χ^2^*	*OR*(95%*CI*)	*P*
A^1^C^2^T^3^G^4^	3(3.0)	3(3.5)	0.06	0.82(0.17,4.00)	0.81
A^1^C^2^T^3^T^4^	86(86.0)	63(66.5)	8.89	**3.16(1.45,6.87)**	**<0.01**
G^1^C^2^C^3^G^4^	4(4.0)	5(5.4)	0.25	0.71(0.18,2.72)	0.62
G^1^C^2^T^3^G^4^	3(3.0)	10(10.4)	4.61	**0.25(0.07,0.96)**	**0.03**
G^1^C^2^T^3^T^4^	1(1.0)	7(7.7)	5.60	**0.12(0.02,0.96)**	**0.02**

1 rs1057079 site,

2 rs17036508site,

3 rs2536 site,

4 rs2295080 site

**Table 6:** Haplotype analysis of the *NOTCH1* gene in subjects

Haplotype	PD+SD (%)	PR(%)	*χ^2^*	*OR*(95%*CI*)	*P*
C^1^A^2^	90(75.2)	83(78.0)	0.23	0.85(0.45,1.63)	0.63
C^1^G^2^	11(8.9)	10(9.7)	0.04	0.91(0.37,2.24)	0.84
T^1^A^2^	16(13.1)	10(9.7)	0.63	1.40(0.61,3.23)	0.42

1 rs3124591 site,

2 rs10521 site

### Correlation of the curative effect of paclitaxel plus cisplatin chemotherapy with advanced ESCC

**Figure 1:**
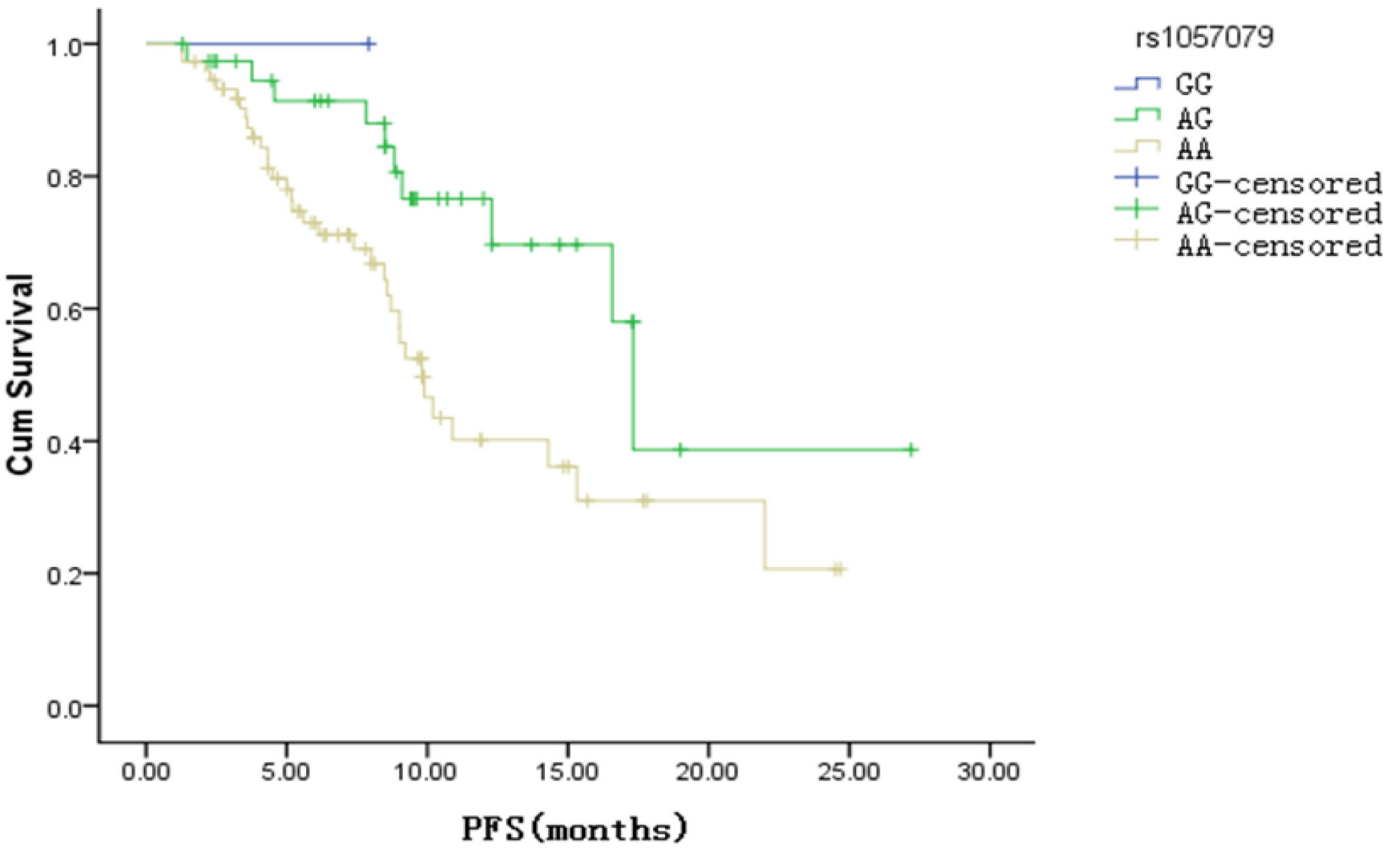
Kaplan Meier survival curve of progression free survival (PFS) about rs1057079 site (p <0.01)

The result of the Kaplan-Meier survival analysis revealed that the median PFS of patients with advanced ESCC who received TP as first-line treatment is 14.3 months (95% CI: 9.0-19.60 months). The association between the genotype distributions of the 11 selected SNPs of *FBW7* and its substrate genes and the PFS was analyzed. The data showed that patients with *mTOR* rs1057079 AG genotype had longer PFS when compared with AA genotype after adjustment for sex, age, and chemotherapy cycle. Only 1 patient was GC genotype in *mTOR* rs1057079. The median PFS of AG genotypes and AA genotypes in *mTOR* rs1057079 was 17.31 months (95% CI: 15.9-18.67 months) and 9.8 months (95% CI: 8.58-11.02 months) (*p* = 0.019), respectively (Figure 1).

## DISCUSSION

Paclitaxel plays a key role in advanced ESCC first-line chemotherapy. However, its clinical utility is limited by drug resistance, as well as largely varied clinical response. Up to now, there is no biomarker that could be used to predict the clinical efficacy of paclitaxel. Notably, recent studies have demonstrated that FBW7 is involved in the regulation of drug resistance [10, 30]. FBW7, a well-studied SCF ubiquitin ligase, is reported to target various oncogenic proteins for ubiquitination [31]. Study has shown that loss of FBW7 leads to an elevated expression of the c-Jun, c-Myc, and Notch-1 oncoproteins, all of which are capable of promoting cell growth [32]. Tumors with mutant FBW7 were more resistant to paclitaxel compared to FBW7 wild-type parental tumors [10]. To our knowledge, this work is the first study to investigate associations between genetic polymorphisms of *FBW7* and its substrate genes and the clinical response of paclitaxel.

We detected *Myb* rs210940, rs7435589, *mTOR* rs1057079, rs17036508, rs2536, rs2295080, *NOTCH* rs3124591, rs10521, *c-Myc, SREBF2 and FBW7* genotypes. Our results demonstrate that patients with *mTOR* rs1057079 AG (OR_adjusted_: 4.59; 95% CI: 1.78-11.86) genotype have significant correlation with the efficacy of paclitaxel when compared with AA genotype after adjustment for sex, age, and chemotherapy cycle. Patients with *mTOR* rs1057079 AG genotype experienced an ORR of 47.2% with the mPFS of 17.3 months (95% CI: 15.9-18.67 months). However the mPFS of patients with *mTOR* rs1057079 AA genotype was 9.8 months (8.58-11.02 months). No significant correlations were observed between the other 10 genotypes examined and the clinical response of paclitaxel. We further analyzed haplotype gene polymorphisms of mTOR. As a result, haplotype of A_rs1057079_C_rs17036508_T_rs2536_T_rs2295080_ can increase the risk of an infaust efficacy of paclitaxel [*OR* (95%*CI*): 3.16(1.45-6.87)]. On the contrary, haplotypes of G_rs1057079_C_rs17036508_T_rs2536_G_rs2295080_and A_rs1057079_C_rs17036508_T_rs2536_T_rs2295080_ can decrease the risk of an infaust efficacy of paclitaxel [*OR* (95%*CI*): 0.25 (0.07-0.96) and *OR* (95%*CI*): 0.12 (0.02-0.96)]. Furthermore, we investigated the association of *FBW7* and *its* substrate gene polymorphisms with situations of adverse events. The data demonstrated that patients with *myb* rs7435589 AG genotype and rs10521 AG type in *NOTCH1* had high risk of grade 3-4 adverse events after receiving paclitaxel plus cisplatin chemotherapy.

The activation of mTOR is known to positively regulate protein translation and cell proliferation. It has been documented that mTOR plays a critical role not only in cancer angiogenesis, but also in cancer progression [33–34]. Two studies have assessed associations between functional SNPs in mTOR gene and risk of ESCC in Chinese populations. They genotyped several mTOR SNPs in ESCC patients and found a significantly altered risk of ESCC associated with*mTOR* rs1883965 and *mTOR* rs2295080 [35–36]. An early study identified seven SNPs of *PI3K/PTEN/AKT/mTOR* pathway in patients with advanced ESCC treated with 5-Fu, cisplatin or paclitaxel, and explored the association between clinical outcome and genetic variations. Only *mTOR FRAP1:rs11121704* homozygosity was associated with a poor response to paclitaxel [37]. Gratifyingly, our work unveiled significant association between genetic variants in *mTOR* and the clinical response of paclitaxel in patients with ESC, although it is too eary for us to conclude that *mTOR* rs1057079 AG is the biomarker of paclitaxel response since the sample sizes of this study is relatively small.

Currently, we are enrolling patients in a large sample size clinical trial to further verify the association between SNPs of *mTOR* and the response of paclitaxel. The ultimate goal is to allow for the selection of the optimal therapy that would provide the most benefit and least toxicity for patients with ESCC from paclitaxel treatment.

To conclude, in SNPs of *FBW7* and its substrate genes, the *mTOR* rs1057079 AG genotype could be used to predict the clinical response and to achieve better mPFS of patients with advanced ESCC treated with TP regimen as first-line chemotherapy. Further investigation with a larger number of patients study is needed to confirm the predictive values of genetic polymorphisms in mTOR signaling pathways.

## MATERIALS AND METHODS

### Patients

This was a prospective analysis of patients with advanced ESCC who received TP as first line chemotherapy. The study was approved by the Ethics Committee of Zhengzhou University. Written informed consents for chemotherapy were obtained from all patients. All patients agreed to these studies and the preservation of genomic DNA for future investigations. Patients had to be at least 18 years of age at the time of registration and had histologically or cytologically confirmed ESCC, which was surgically unresectable or recurrent. They also had to have an Eastern Cooperative Oncology Group (ECOG) performance status of 0–2, a life expectancy of >12 weeks with sufficient bone marrow, liver, renal and cardiovascular function.

### Treatment plan

Patients were treated with paclitaxel 175 mg/m^2^ over 3 hours day 1 and cisplatin 75 mg/m^2^ day 1, every 3 weeks. Patients receiving paclitaxel were premedicated with 10 mg of oral dexamethasone at 12 and 6 hours before the infusion and with intramuscular injection diphenhydramine and intravenous H2 receptor antagonist within 60 minutes before the infusion of paclitaxel to reduce the risk of hypersensitivity reaction. Cisplatin was administered with hydration and forced diuresis, and patients underwent routine monitoring of electrolytes, serum creatinine, and magnesium. The combination therapy was up to maximum 6 cycles. Treatment was discontinued until the documented disease progression, unacceptable toxicity or patient's refusal.

### SNP selection and genotyping

*Myb, mTOR, NOTCH, c-Myc, SREBF2* and *FBW7* genes were identified based on Chinese population data in HapMap database (http://www.hapmap.org). SNPs in *Myb, mTOR, NOTCH, c-Myc, SREBF2* and *FBW7* were selected by bioinformatics data bank using Haploview software, bioinformatics software packages such as TargetScan Human 6.2 (http://www.targetscan.org/), miRanda (http://microrna.org/) and Patrocles (http://www.patrocles.org/) to offer potential miRNA binding sites within the 3′UTRs of these genes. As a result, totally 11 SNPs in these genes were included for further genotyping with the criteria of a minor allele frequency (MAF) greater than 5%. Polymerase chain reaction restriction fragment length polymorphism (PCR-RFLP) was used to analyze the polymorphisms of *mTOR* rs1057079, rs17036508, rs2536, rs2295080, *NOTCH* rs3124591, rs10521, *c-Myc, SREBF2 and FBW7* rs7685296. The digestion products were visualized by electrophoresis on 3% agarose gel and the genotypes were inferred from the number of bands observed in the gel.

### Statistical analysis

Response was assessed according to the Response Evaluation Criteria in Solid Tumors (RECIST) version 1.0 [28] as complete response (CR), partial response (PR), stable disease (SD) or progressive disease (PD) in patients with measurable lesions. Toxicities were graded according to the National Cancer Institute's Common Terminology Criteria for Adverse Events (CTCAE) version 4.0 [29]. Progression-free survival (PFS) was measured from the initiation of TP to the occurrence of progression, or death without evidence of progression. Follow-up evaluations were performed after every 3 months for 3 years by endoscopy and CT scan. The last follow-up was performed in June 2015.

The SPSS 21.0 statistical software package was used for statistical analyses. Student's t-test for continuous variables and Chi-squared (*c^2^*) test for categorical variables were used to compare the differences in the distributions of major demographic variable, as well as the genotypes of selected SNPs between the ESCC patients. Unconditional logistic regression was applied to estimate the associations between genetic polymorphisms and the risk of ESCC by computing the odds ratios (ORs) and its 95% confidence intervals (CIs). Online SHEsis (http://analysis.bio-x.cn/myAnalysis.php) was applied for haplotype prediction and analysis. The Kaplan-Meier method was used to estimate the relationship between PFS and clinical response of paclitaxel; the log-rank test of the null hypothesis of a common survival curve was used to compare survival curves. A P-value of less than 0.05 was considered to be statistically significant and all the inspections were two-side test.
